# Postprandial metabolic responses of serum calcium, parathyroid hormone and C-telopeptide of type I collagen to three doses of calcium delivered in milk

**DOI:** 10.1017/jns.2014.2

**Published:** 2014-04-30

**Authors:** Marlena C. Kruger, Pamela R. von Hurst, Christine L. Booth, Barbara Kuhn-Sherlock, Joanne M. Todd, Linda M. Schollum

**Affiliations:** 1 Institute of Food, Nutrition and Human Health, Massey University, Private Bag 11222, Palmerston North, New Zealand; 2 Institute of Food, Nutrition and Human Health, Massey University, Private Bag 102 904, North Shore Mail Centre, Auckland, New Zealand; 3 Fonterra Research and Development Centre, Private 11029, Palmerston North, 4442, New Zealand; 4 Fonterra Co-Operative Group Ltd, Private Bag 92032, 9 Princes Street, Auckland, New Zealand

**Keywords:** Calcium absorption, Milk, Asian women, Parathyroid hormone, Urinary calcium, C-telopeptide of type I collagen, CTx, C-telopeptide of type I collagen, PTH, parathyroid hormone

## Abstract

Acute doses of Ca rapidly increase serum Ca and reduce bone resorption concomitant with a reduction in serum parathyroid hormone (PTH) levels. The physiological response to a dose of Ca in milk and to a Ca salt may be different. The present study investigated Ca absorption patterns with increasing levels of fortification in milk, and the response to one dose of a Ca salt. A group of twenty-eight Asian women aged 20–45 years volunteered to attend the laboratory over several weeks. The fasted volunteers were randomised to one of three experimental drinks: 200 ml skimmed milk containing 250, 500 or 1000 mg Ca. A subgroup of seven volunteers also received a calcium gluconate/carbonate salt containing 1000 mg Ca in 200 ml water. Serial blood samples and urine were collected for 5 h from baseline. Different doses of Ca in milk resulted in a graded response in serum corrected Ca, PTH and C-telopeptide of type I collagen (CTx) but not ionised Ca. Serum Ca increased in response to all milk drinks and from 2 to 5 h the blood Ca levels were significantly different for the 250 and 1000 mg doses, as was the integrated response between the loads. The PTH response to the two higher doses was significantly more than following the 250 mg dose. The integrated response for CTx and urinary Ca between all three doses of Ca in milk was significantly different. A dose of Ca salt elicited a more immediate response reaching a plateau faster, and declining faster to baseline. Fortified milk is a safe matrix for delivering larger doses of Ca.

Adequate Ca intake is important to achieve optimal peak bone mass and prevent or reduce bone loss with ageing. Obtaining the recommended daily Ca allowance from foods is recommended, as other bioactives could be present that may enhance the health benefits of the food. Milk can be fortified with a Ca salt to increase the amount of Ca delivered per dose of milk and Ca from milk is usually well absorbed.

An acute oral dose of a Ca salt rapidly increases serum corrected, i.e. Ca corrected for albumin, as well as ionised Ca and reduces bone resorption (serum C-telopeptide of type I collagen; CTx) with a concomitant reduction in parathyroid hormone (PTH) levels^(^^1^^–^^4^^)^. Fractional Ca absorption is an inverse function of load size^(^^5^^–^^7^^)^. Measuring the response to three doses of a Ca salt, using urinary Ca and serum Ca concentrations, indicated that there is a plateau in the postprandial response at a dose of 500 mg with a slight increase in the response to a dose of 1000 mg^(^^8^^,^^9^^)^. Heaney *et al*.^(^^7^^)^ reported that the Ca response slopes for a single dose of 1 g calcium carbonate *v.* divided doses over several hours were similar up to approximately 5 h and that absorption was capacity limited even when the doses were divided. In addition, Karkkainen *et al*.^(^^10^^)^ reported a significant difference between the changes in serum ionised Ca induced by a dose of 250 mg compared with 1000 mg Ca.

An acute dose of Ca in milk, however, results in a slower change in serum ionised Ca in comparison with a similar dose of a Ca salt^(^^11^^–^^13^^)^. Karkkainen *et al*.^(^^14^^)^ compared the postprandial Ca response from four different foods including milk, and showed that ingestion of 400 mg Ca in milk caused a significantly smaller increase in serum ionised Ca compared with the control Ca salt. Other studies report similar findings when a Ca salt was compared with milk or Ca-fortified milk^(^^11^^)^, confirming that the matrix in which the Ca is delivered could therefore affect the rate of appearance and the rise in serum Ca.

More recent studies using milk as the vehicle showed that an acute dose of Ca (1200 mg per dose) in milk also suppresses both PTH levels and bone resorption in adult men and women^(^^11^^,^^15^^,^^16^^)^. When milk fortified with milk Ca was compared with milk fortified with calcium carbonate at a single dose of 1200 mg Ca, the calcium carbonate-fortified milk suppressed PTH and bone resorption significantly more than the milk fortified with milk Ca^(^^11^^,^^13^^)^.

In longer-term studies, Kruger *et al*.^(^^17^^,^^18^^)^ showed that the reduction in PTH and CTx resulting from supplementing pre- and postmenopausal women with 1200 mg Ca per d as fortified milk was maintained over the 12–16 weeks of the interventions.

The objectives of the present study were to assess the acute postprandial physiological response to a series of different Ca loads in a milk-based drink, by measuring serum PTH, serum CTx, serum corrected and ionised Ca and urinary Ca excretion over a 5 h period. A secondary objective was to compare the postprandial response to a similar load of Ca as a salt in water or as a salt in milk.

## Methods

The present study was conducted according to the guidelines laid down in the Declaration of Helsinki and all procedures involving human subjects were approved by the Massey University Human Ethics Committee (Southern A; approval no. 10/65). Written informed consent was obtained from all subjects. The study was run as a single-blinded randomised controlled trial.

### Experimental subjects

A total of twenty-eight Asian female volunteers aged 20–45 years, living in Auckland or Palmerston North, New Zealand, were recruited from the community through the local newspapers and Massey University Campus advertising. Inclusion criteria were aged 20–45 years, the ability to tolerate a glass of milk, and not taking Ca supplements or vitamin D supplements, or, if so, agreeing to stop these for 4 weeks before entering the trial. Other exclusion criteria were more than three units of alcohol per d, smoking, endocrine disease, diagnosis of any form of cancer, vascular disease or diabetes mellitus. The trial was preceded by a health screen of liver and kidney function and haematology.

### Anthropometry

Body weight was measured using a digital balance (model BWB-627-A; Tanita Corporation) to the nearest 0·1 kg, and standing height was measured using a stadiometer (Institute of Fundamental Sciences, Engineering Services Workshop, Massey University) to the nearest 0·1 cm. Bone mineral density of the total hip, femoral neck and lumbar spine was measured using a Hologic Discovery A densitometer.

### Food intake

Food intake was assessed using a 3 d recall at baseline. Dietary macro- and micronutrient composition was calculated from the New Zealand Food Composition Table, accessed using nutrient analysis software (FOODworks 2009; Xyris software (Australia) Pty Ltd).

### Procedures

Volunteers attended the Human Research Laboratories at Massey University campuses at Palmerston North or Albany on three occasions at least 1 week apart, for 7 h at each occasion. They were randomised by the study nurse (computer-generated random numbers) to ingest one of three milk drinks at each occasion. The volunteers arrived in the laboratory at approximately 08.30 hours after fasting since 21.00 hours the previous night. The volunteers were asked to empty their bladders. An indwelling intravenous cannula was placed in an antecubital vein and kept patent using normal saline. Baseline blood samples were taken shortly after the cannula was sited. The volunteers were then given one of the three test drinks, which they consumed within 15 min. In order to avoid any effect of circadian rhythm in the measurements, the volunteers were asked to begin consuming the drinks between 09.00 and 09.30 hours; the actual time was recorded and referred to as time zero. The blood sampling times were staggered so that the time from taking the test drink to the time that the first blood sample was taken was exactly the same for each participant.

The milk drinks contained either 250 mg Ca (20·1 g skimmed milk powder; NZMP™ Fonterra Ingredients Ltd), 500 mg Ca (30 g fortified reduced fat milk powder; Anlene™, Fonterra Brands Ltd) or 1000 mg Ca (30 g fortified reduced fat milk powder; Fonterra Brands Ltd) in 200 ml of deionised water followed by another 50 ml to wash out the container of any residual milk drink. Blood samples (20 ml) were collected every 1 h, and urine was collected every 2 h period for 6 h. The volunteers were permitted 100 ml water every 1 h.

The blood samples were kept cold until they were spun at 2000 ***g*** for 10 min at 4°C and the supernatant fraction was sampled and stored frozen until analysis. Urine was collected after the first morning void until time zero, and was then collected from baseline every 2 h for 6 h from the start of consuming the drink. The urine volumes were measured, and a representative sample was then stored frozen until analysis.

The volunteers were given two plain biscuits after 2 h and a light lunch after 4 h, comprising two slices of wholemeal bread, thinly spread with sunflower margarine, and 130 g canned peaches in syrup. The composition of the food that was consumed during the day is shown in Table 1.

**Table 1.** Nutrient composition of the single meal consumed during each 1 d study

Nutrient	Amount	% Energy intake
Energy (kJ)	1510	N/A
Carbohydrate (g)	64·1	68
Protein (g)	8·2	9·2
Fat (g)	8·5	20·8
Ca (mg)	35·2	N/A
Vitamin D (μg)	0·31	N/A

N/A, not applicable.

A subgroup of seven women attended the human studies laboratory in Palmerston North twice more. On these occasions they received either the fortified milk with 1000 mg Ca or 1000 mg Ca from a tablet containing 2·3 g calcium lactate gluconate and 1·8 g calcium carbonate (Calsource Ca1000; Novartis) in 200 ml water in a random order. The experimental procedure was the same as above.

### Primary outcome measures

Serum and urinary Ca was measured by spectrophotometry using the Arsenazo-III dye reaction with an Abbott c8000 analyser (Abbott Laboratories). P was measured using the phosphomolybdate method, and albumin was measured using the bromcresol green method on the Abbott c8000 analyser. Serum Ca was corrected for albumin. Ionised Ca was measured using a Ca electrode (ion sensitive) and a potentiometric method using the Nernst equation. All the above analyses were performed by Medlab Central, Palmerston North, or Lab Plus, Auckland, New Zealand.

Serum 25-hydroxyvitamin D was measured using isotope-dilution liquid chromatography–tandem MS^(^^19^^)^. The CV for the vitamin D assays ranged from 6·8 to 10·2 %. Serum cross-linked CTx (CrossLaps) and PTH were measured by electrochemiluminescence immunoassays using the Roche Elecsys 2010 system and commercially available kits (Roche Diagnostics, GmbH). For CTx the uncertainty of measurement was between 4·6 and 5·3 % depending on the level of CTx, and for PTH was between 3·1 and 5·6 % depending on the level of PTH measured. These assays were performed by Canterbury Health Laboratories, an accredited clinical facility.

### Statistical analyses

Outcome variables (AUC, raw data and difference from baseline) were analysed using linear mixed-model ANOVA (Proc Mixed, SAS version 9.1; SAS Institute Inc.). Integrated AUC for the change from baseline (1 to 5 h) were calculated for each serum measurement. The model included the effects of subject, treatment, visit and baseline concentration.

For the repeated-measures analysis, subject, treatment, visit and time were included in the procedure, in addition to the treatment × time interaction which addresses whether the trajectory over time during the visit differs between treatments (treatment × time). The model for difference from baseline included the result at baseline as a covariate, but only five time points (1, 2, 3, 4 and 5 h).

Where the linear mixed-model ANOVA was significant, Tukey's *post hoc* analysis was used for comparisons between treatments. Missing data were assumed to be missing at random and no data imputation was performed. Statistical significance was set at a level of *P* ≤ 0·05. Efficacy endpoints (AUC, difference from baseline, or raw data) are presented as least-squares means (lsmeans) and 95 % CI.

## Results

### Physical characteristics of the volunteers

A total of twenty-eight women of Asian ethnicity were recruited in Palmerston North (*n* 13) and Auckland (*n* 15). The majority of the women reported their ethnicity as Chinese (*n* 19), together with five Malays, three from Thailand and one from the Philippines. Their baseline characteristics and bone density results are shown in Table 2.

**Table 2.** Baseline characteristics of the study population (Mean values, standard deviations and ranges; *n* 28)

	Mean	sd	Range
Age (years)	26·7	4·3	20 to 37
BMI (kg/m^2^)	21·2	2·3	16·7 to 26·8
Lumbar spine BMD (g/cm^2^)	0·979	0·106	0·733 to 1·206
Lumbar spine *Z* score	−0·5465	0·966	−2·8 to 1·5
Femoral neck BMD (g/cm^2^)	0·813	0·109	0·650 to 1·064
Femoral neck *Z* score	−0·2786	0·973	−1·8 to 2·0
Total hip BMD (g/cm^2^)	0·884	0·104	0·712 to 1·154
Total hip *Z* score	−0·4071	0·882	−1·8 to 1·8
Serum Ca-corrected (mmol/l)	2·23	0·51	2·13 to 2·31
Serum ionised Ca (mmol/l) (*n* 13)	1·12	0·02	1·09 to 1·16
Urinary Ca:creatinine ratio	0·2306	0·102	0·0333 to 0·4800
Serum 25(OH)D (nmol/l)	43·6	18·9	14·0 to 99·0
Parathyroid hormone (pmol/l)	4·519	1·216	2·43 to 7·33
Serum P (mmol/l)	1·256	0·086	1·080 to 1·430
CTx (μg/l)	0·362	0·119	0·123 to 0·753

BMD, bone mineral density; 25(OH)D, 25-dihydroxyvitamin D; CTx, C-telopeptide of type I collagen.

### Biochemical analysis

The baseline biochemical measurements are presented in Table 2. Vitamin D status was negatively associated with baseline PTH levels (*r* –0·448; *P* ≤ 0·001) and with baseline CTx levels (*r* 0·219; *P* = 0·046). Baseline PTH levels were significantly correlated with baseline serum corrected Ca (*r* –0·373; *P* < 0·05) and baseline CTx (*r* –0·217; *P* < 0·047). Dietary Ca intake was associated with baseline PTH (*r* –0·243; *P* < 0·025), change in urinary Ca (*r* –0·266; *P* < 0·015), change in PTH (*r* 0·365; *P* < 0·001) and change in CTx (*r* 0·224; *P* < 0·04). Using 50 nmol/l as the level for vitamin D adequacy, about 21 % of the women were vitamin D deficient, 50 % insufficient and 28 % had adequate vitamin D levels.

### Dietary analysis

Dietary intake was calculated from a 3 d estimated food diary completed by the subjects, and relevant average intakes are listed in Table 3. The mean Ca intake was 587 mg/d, with a range between 189 and 1440 mg.

**Table 3.** Dietary nutrient intake from 3 d food diaries (Mean values, standard deviations and ranges; *n* 28)

	Mean	sd	Range
Total energy (kJ)	5909	1748	3002–10 329
Carbohydrate (g)	159·3	54·9	78·7–267·3
Protein (g)	71·2	23·7	27·1–118·0
Total fat (g)	55·7	24·8	17·0–133·4
Ca (mg)	587	335	189–1440
Mg (mg)	234	97	86–451
P (mg)	1040	387	359–1694

### Serum calcium

**Fig. 1.**
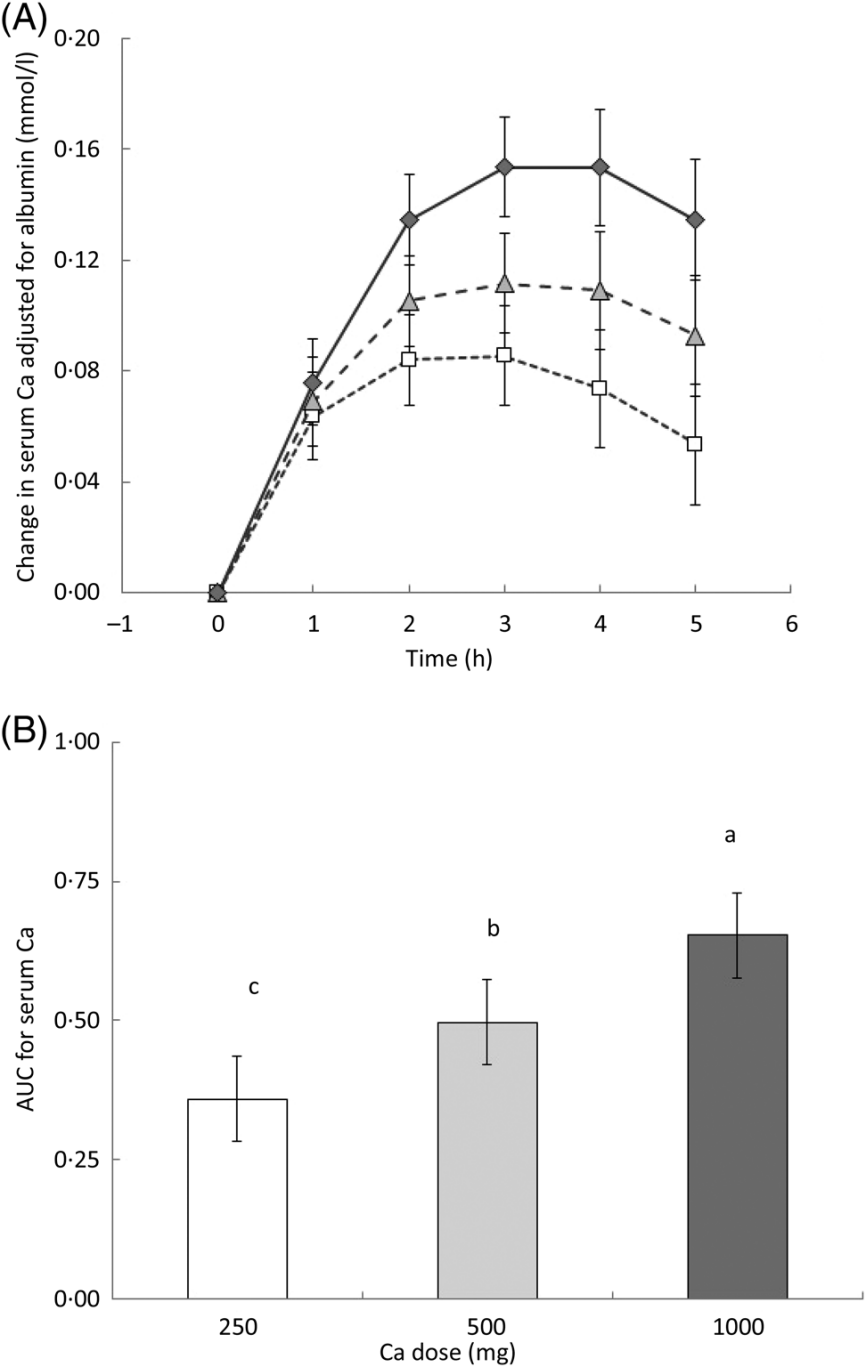
(A) Change in serum calcium adjusted for albumin from baseline over time for each milk drink: 250 mg dose (□); 500 mg dose (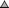); 1000 mg dose (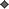). Values are means, with 95 % CI represented by vertical bars. There was an effect of drink × time (*P* = 0·005). (B) AUC for serum calcium concentration over the 5 h period. Values are means, with 95 % CI represented by vertical bars. ^a,b,c^ Mean values with unlike letters were significantly different (*P* < 0·001).

The level of Ca in the drink combined with the influence of time (drink × time) had a significant effect on serum total Ca adjusted for albumin (*P* < 0·005). Serum Ca increased in response to all milk drinks in the first 3 h following consumption (Fig. 1(A)). There was a significant difference in serum Ca concentration between the 250 mg dose and the 1000 mg dose from time point 2 h to 5 h. At time point 3 h, serum Ca was significantly higher for the 1000 mg dose compared with the 500 mg dose. Between 3 and 4 h serum Ca reached a plateau when the 1000 and 500 mg drinks were consumed, but began to decline after the fourth hour. However, following consumption of the 250 mg drink, serum Ca began to decline from the third hour. There was a significant difference in the integrated responses (AUC) for serum Ca between the 250 and 500 mg loads (*P* = 0·01), the 500 and 1000 mg (*P* = 0·003) and between the 250 and 1000 mg (*P* < 0·001) loads (Fig. 1(B)).

**Fig. 2.**
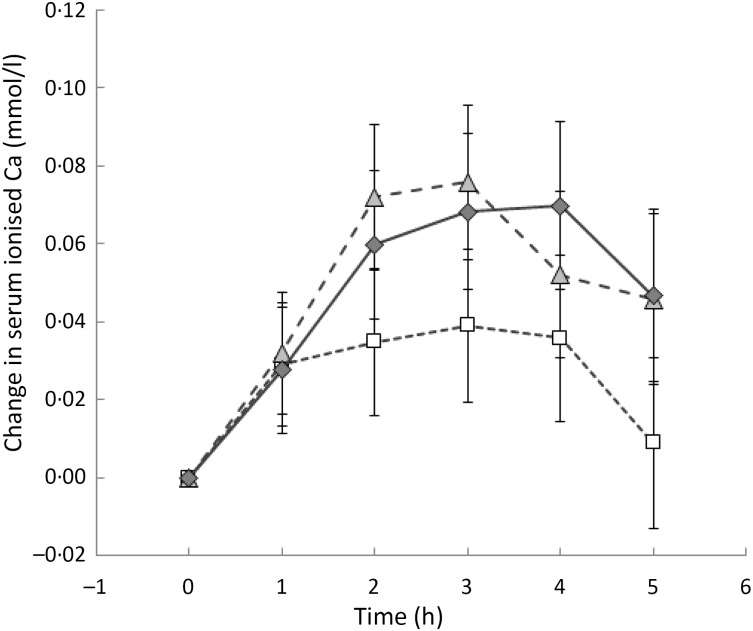
Change in serum ionised calcium from baseline over time for each milk drink: 250 mg dose (□); 500 mg dose (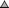); 1000 mg dose (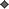). Values are means, with 95 % CI represented by vertical bars.

Serum ionised Ca was measured in a subset of thirteen subjects. There was no significant difference at any one time between the three milk drinks (Fig. 2). However, there was a trend for the AUC for the 250 mg drink to be less than for the 500 mg drink (*P* = 0·064) (data not shown).

### Parathyroid hormone

**Fig. 3.**
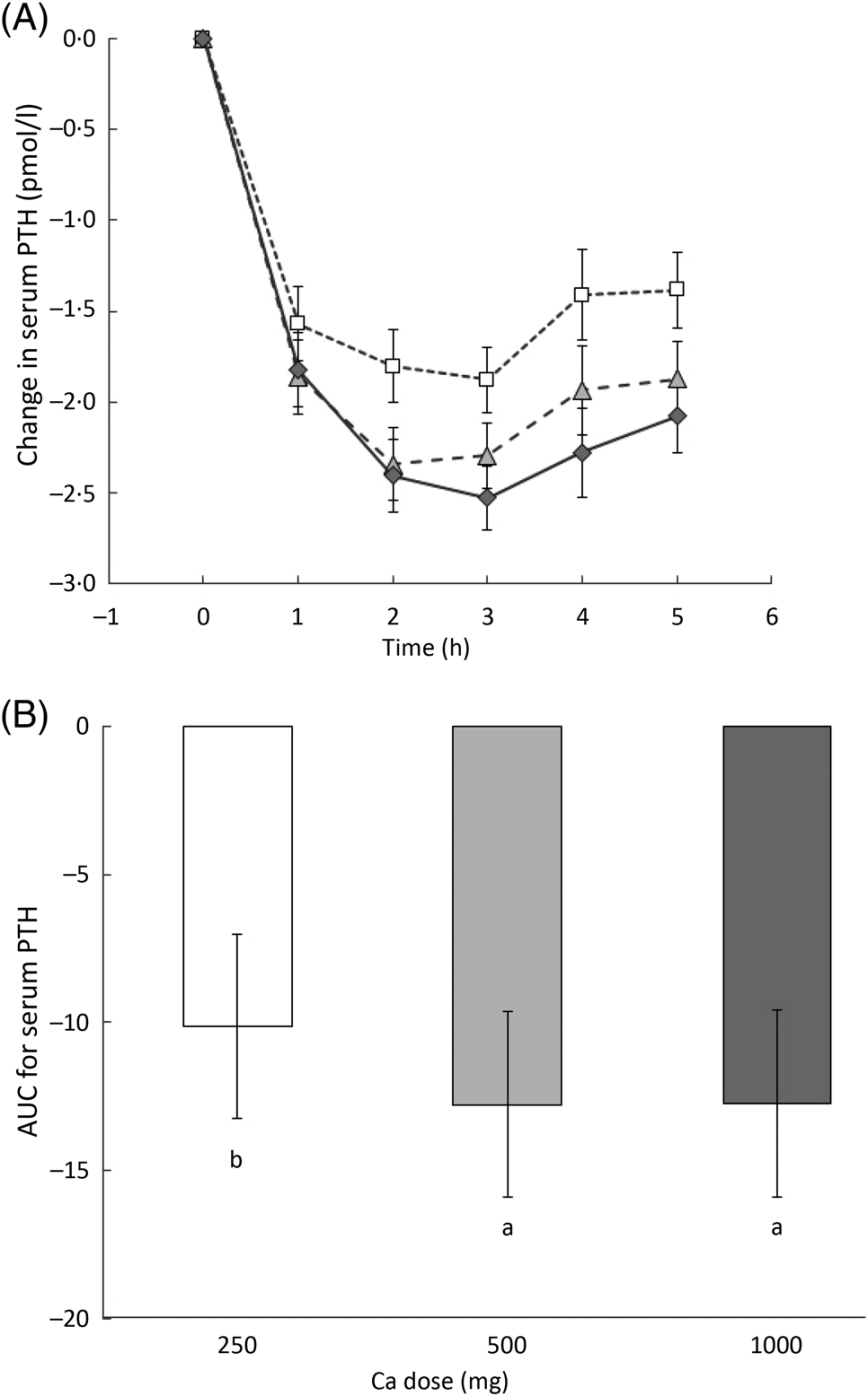
(A) Change in serum parathyroid hormone (PTH) from baseline over time for each milk drink: 250 mg dose (□); 500 mg dose (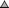); 1000 mg dose (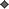). Values are means, with 95 % CI represented by vertical bars. (B) AUC for change in serum PTH from baseline to end point. Values are means, with 95 % CI represented by vertical bars. ^a,b^ Mean values with unlike letters were significantly different (*P* < 0·001).

There were significant treatment (*P* < 0·001) as well as time (*P* < 0·001) effects on the PTH levels, but the interaction was borderline significant (*P* = 0·068). Serum PTH concentration decreased markedly between baseline and 1 h in response to all the milk drinks. From 2 to 5 h, PTH concentrations remained significantly lower in response to the 500 and 1000 mg drinks compared with the 250 mg drink (Fig. 3(A)). There was a significant difference in the integrated responses between the 250 and the 500 mg (*P* < 0·001) as well as between the 250 and the 1000 mg dose (*P* < 0·001) (Fig. 3(B)). There was no significant difference between the integrated responses to the 500 *v.* the 1000 mg dose.

### Serum C-telopeptide of type I collagen

**Fig. 4.**
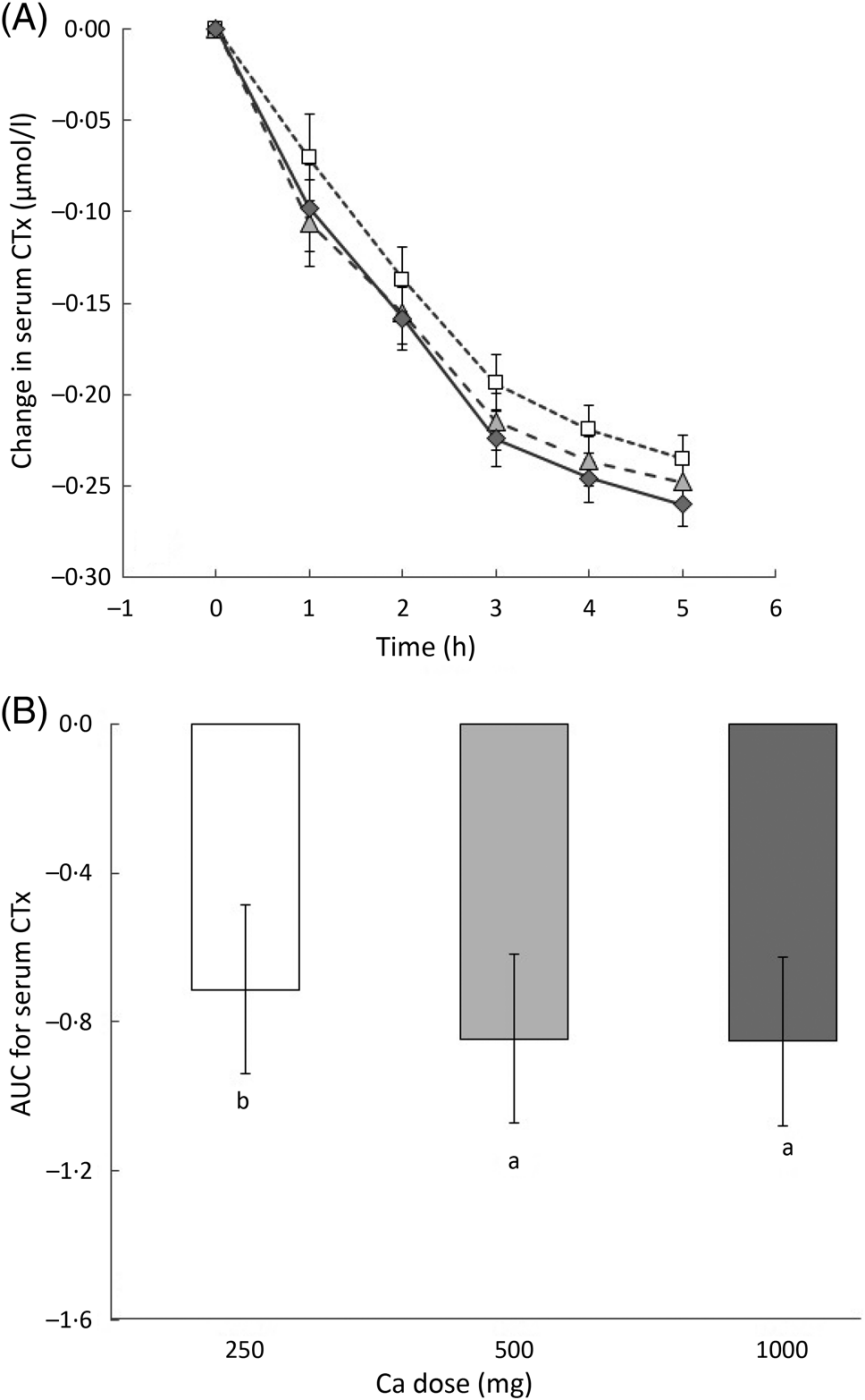
(A) Change in serum C-telopeptide of type I collagen (CTx) over time for each milk drink: 250 mg dose (□); 500 mg dose (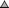); 1000 mg dose (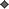). Values are means, with 95 % CI represented by vertical bars. (B) AUC for change in serum CTx from baseline to end point. Values are means, with 95 % CI represented by vertical bars. ^a,b^ Mean values with unlike letters were significantly different (*P* = 0·001).

There were significant treatment and time effects on CTx levels but no significant interaction between these. Serum CTx levels decreased steadily from baseline throughout the 5 h of the trial (Fig. 4(A)). From time point 4 and 5 h, there was a significant difference between changes in CTx for the 250 *v.* the 1000 mg dose (*P* = 0·002). There was significant difference in the integrated response between the 250 mg dose and the 500 mg as well as the 1000 mg dose (*P* < 0·001) (Fig. 4(B)).

### Urinary calcium

**Fig. 5.**
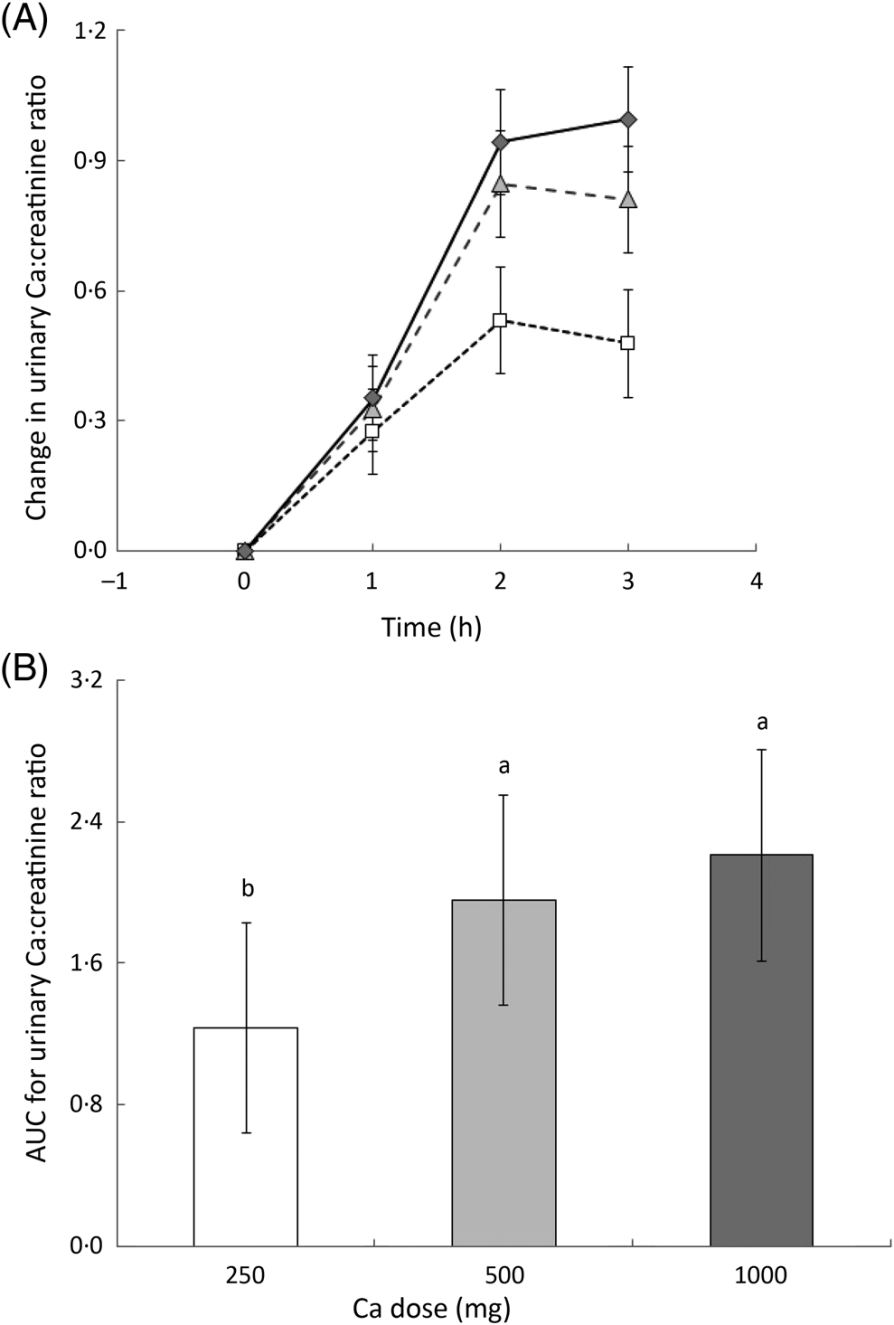
(A) Urinary calcium:creatinine ratio differences from baseline as measured at three time points during 6 h for each milk drink: 250 mg dose (□); 500 mg dose (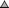); 1000 mg dose (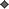). Values are means, with 95 % CI represented by vertical bars. (B) AUC for change in urinary calcium:creatinine ratio from baseline to end point. Values are means, with 95 % CI represented by vertical bars. ^a,b^ Mean values with unlike letters were significantly different (*P* < 0·021).

Urinary Ca excretion was measured at baseline and then in 2 h intervals and is reported as the urinary Ca:creatinine ratio. Urinary Ca in relation to creatinine increased 2-fold in the first 2 h following consumption of all the milk drinks. By the second urine collection at 4 h, the ratio was significantly greater (*P* < 0·001) following consumption of the 500 and 1000 mg drinks than following the 250 mg drink (Fig. 5(A)). At the final urine collection (6 h) the ratio was decreasing in response to the 250 and 500 mg drinks, but continuing to increase in response to the 1000 mg drink. At this time point the ratio was still significantly higher following the 500 and 1000 mg drinks compared with following the 250 mg drink.

There was significant difference in the integrated response between the 250 mg dose and the 500 mg as well as the 1000 mg dose (*P* < 0·021) (Fig. 5(B)).

**Fig. 6.**
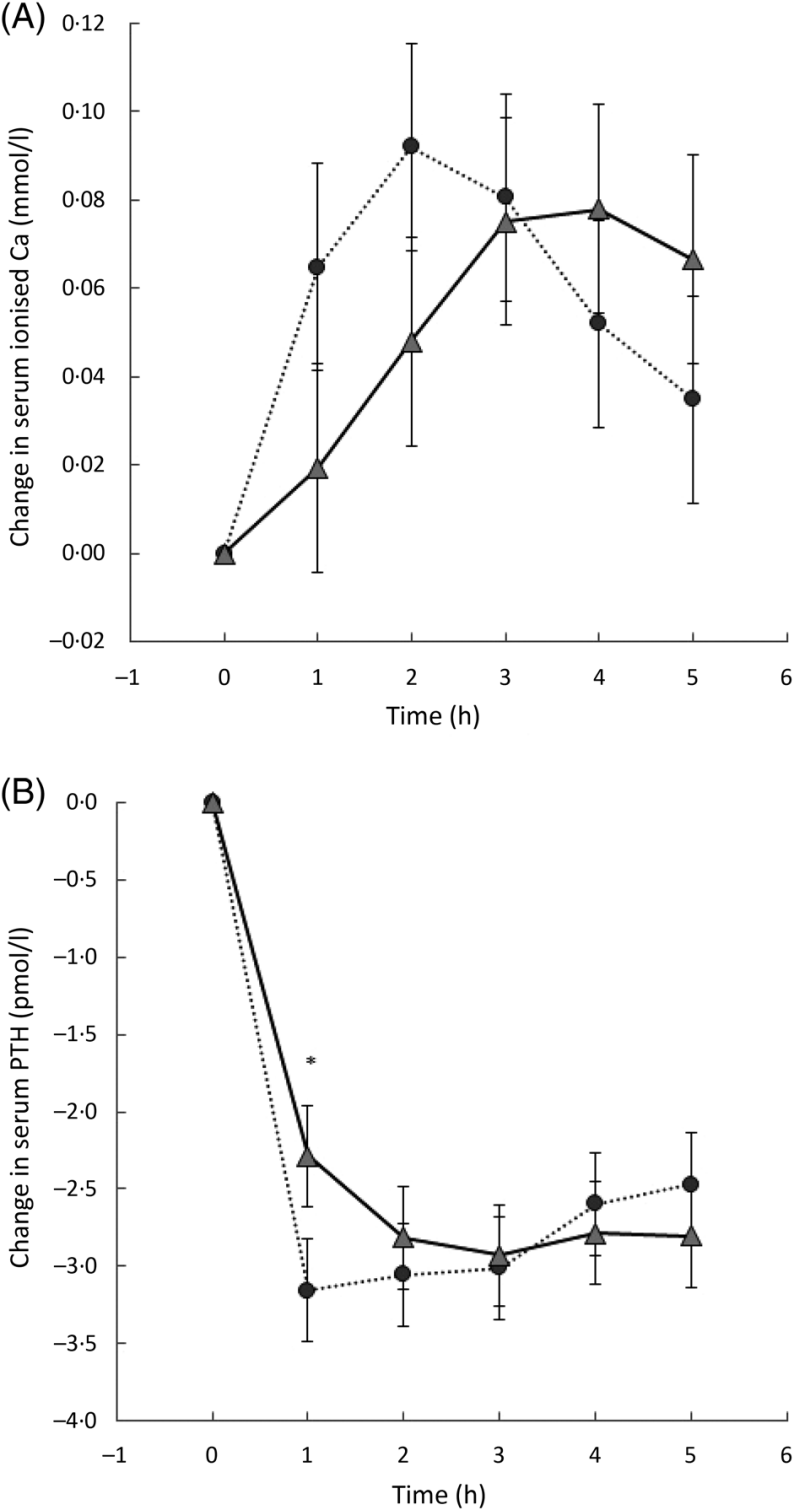
(A) Change in serum ionised calcium from baseline over time for the tablet containing 1000 mg calcium (•) or the milk delivering 1000 mg calcium (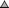). Values are means, with 95 % CI represented by vertical bars. (B) Change in serum parathyroid hormone (PTH) levels over time after a dose of 1000 mg as the salt (•) or in milk (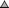). Values are means, with 95 % CI represented by vertical bars. * Mean value was significantly different from that for the salt (*P* < 0·05).

Comparison of the response to 1000 mg Ca in milk *v.* the Ca salt showed no difference in serum corrected or ionised Ca, CTx or urinary Ca:creatinine ratio. For serum ionised Ca and PTH, there were interactions between treatment and time, but no overall treatment effect and no effect on AUC. This indicates that there were no differences in the total levels for PTH or Ca measured, but the patterns of appearance/disappearance in the blood over time were different for the two treatments. After the oral dose of the Ca salt, ionised Ca increased faster, reached its peak earlier and decreased sooner and faster than with the Ca dose in milk (Fig. 6(A)). With the dose of Ca salt, serum PTH decreased faster and reached a plateau earlier when compared with the milk group (Fig. 6(B)).

## Discussion

The aim of the present study was to assess the physiological response to three doses of Ca delivered in milk, as well as comparing the response to a Ca salt in water. The data presented show that there is a graded physiological response in total blood Ca concentration depending on the dose of Ca taken. A similar dose of Ca as the salt compared with the dose in milk resulted in a faster rise in ionised Ca in the blood. The peak concentration was reached earlier and the blood levels decreased earlier compared with a Ca dose in milk. As serum Ca increased, PTH responded in an inverse manner, reaching the lowest level after 3 h and then beginning to increase again. The largest response was to the dose of 1000 mg Ca in milk, with a lower response to 500 mg and a significantly lower response to 250 mg. Serum CTx levels decreased steadily over the 5 h with a significant difference between the response to 250 mg and those to 500 or 1000 mg in milk, while there was no difference in the response to 1000 mg Ca as the salt or in milk.

Ca supplementation has been shown in several short- and long-term studies to slow age-related bone loss and to reduce the risk of hip fracture in older men and women. The absorption efficiency of the Ca is important for the beneficial effects on bone. A number of studies have been reported that compared various Ca formulations containing different salts, in short acute absorption trials as well as longer supplementation trials^(^^1^^–^^3^^,^^14^^,^^20^^)^. The data from the acute trials report a rapid increase in serum ionised as well as corrected Ca after an oral dose. The response varies according to type of salt used^(^^2^^,^^9^^,^^21^^–^^23^^)^ as well as the dose of the salt^(^^1^^,^^9^^,^^24^^)^. Guillemant & Guillemant^(^^1^^)^ reported that the change in ionised Ca after a 1500 mg dose of Ca from calcium gluconate/carbonate was significantly higher than when compared with a dose of 500 mg while Zikán *et al*.^(^^24^^)^ reported a more prolonged increase in ionised Ca after a load of 1 g *v.* a load of 0·2 g elemental Ca.

When a food or milk is used as the vehicle for delivering the Ca, the pattern of absorption may be different from the responses reported with a Ca salt using water as the carrier. The absorption of similar levels of Ca from Emmental cheese, milk and spinach was slower compared with the response to calcium lactate gluconate, and serum ionised Ca did not reach the same maximal concentration, although the serum ionised Ca response after consuming cheese almost reached a similar level to that of the Ca salt, though with a 2 h delay^(^^14^^)^. In contrast, Nickel *et al*.^(^^25^^)^ reported that fractional absorption of Ca from various dairy products was similar, and Martini & Wood^(^^26^^)^ reported that a dose of 500 mg Ca from Ca-fortified orange juice, skimmed milk and calcium carbonate did not result in significantly different serum levels of Ca. The acute response to a dose of calcium carbonate or milk Ca in milk as the carrier did, however, elicit a different response, with calcium carbonate raising blood Ca significantly more compared with milk Ca^(^^11^^)^. Comparing regular milk with milk Ca- or tricalcium phosphate-fortified milk, increased absorption of Ca from the fortified milks using stable isotopes was demonstrated^(^^27^^)^. No other investigations could be found where different doses of Ca in milk were compared with each other in the same study. In addition, the present study also showed that the response to a load of Ca from a salt is faster, increasing serum and ionised Ca more rapidly but reaching a plateau quicker, compared with the same dose in milk, in accordance with Green *et al*.^(^^15^^)^ and López-Huertas *et al*.^(^^27^^)^.

The three doses of Ca in milk suppressed serum PTH in a dose-dependent manner but the responses to the 500 and the 1000 mg Ca doses were not significantly different. The suppression of PTH by Ca is a response to maintain Ca homeostasis, and is mediated by the Ca-sensing receptor in the parathyroid gland. This receptor responds to the transient increase in plasma ionised Ca within seconds. Several authors have reported the Ca-dependent postprandial decrease in PTH^(^^1^^,^^3^^,^^11^^,^^13^^,^^14^^,^^26^^,^^28^^)^ and also reported a dose-dependent response^(^^14^^,^^23^^)^ using a variety of foods depending on the bioavailability of Ca from the foods. No reports could be found where the response to various doses of Ca in the same food, or from milk, was compared. In the present study the serum PTH concentration decreased in parallel with the increase in serum and ionised Ca concentrations, the latter affected by the dose-dependent absorption of Ca in the intestine. We did not show a significant difference in the ionised Ca concentration between the various doses but there was a trend for the response after the 250 mg dose to be lower than the response to the 500 and 1000 mg dose. Accordingly, the response by serum PTH was significantly higher for the 500 and 1000 mg doses of Ca compared with the 250 mg dose.

Serum CTx levels decreased from baseline over the 5 h of sampling, and did not reach a plateau by 5 h. The response to the 250 mg dose was significantly less than the response to the 500 and 1000 mg doses between time points 4 and 5 h but all three doses significantly decreased CTx. The reduction in PTH over time suggested that bone remodelling was being suppressed by the various doses of Ca and this was confirmed by the reduction in CTx. PTH decreased rapidly after the dose of Ca, reaching a minimum value by 3 h. In contrast, the response by CTx was slower, and more continuous, and was still strongly suppressed at 5 h after the Ca dose. The delay in response by CTx to a Ca load was also reported by Zikán *et al.*^(^^24^^)^ in young women and by Green *et al.*^(^^11^^,^^15^^)^ and Martini & Wood^(^^26^^)^ in postmenopausal women. The immediate response of PTH to a Ca load may therefore lead to the reduction in CTx as well, albeit slower, suggesting that the suppression of bone resorption may last longer than the actual decrease in serum PTH levels. The osteoclast has also been shown to have Ca-sensing receptors^(^^24^^)^, and Ca ions may therefore directly regulate bone resorption. The response of the osteoclast may, however, only occur at higher extracellular Ca concentrations^(^^24^^)^. The osteocyte is able to regulate small changes in serum Ca by the process of osteocytic osteolysis. This process does not involve bone resorption but only movement of Ca controlled by the osteocyte. This process, however, is short; response is within 1–3 h after a change in serum Ca and does not affect bone resorption and would not elicit a response by PTH or CTx^(^^29^^)^.

In a controlled situation there is a strong correlation between acute intake of Ca and urinary Ca excretion. The excretion of Ca increased as the dose of Ca in milk increased, but the change in excretion was not significantly different between the 500 mg and the 1000 mg dose, while both of these were significantly higher than the excretion after the 250 mg dose. There was, however, a significant difference in the integrated response between the three doses from milk (Fig. 3(B)). Urinary Ca usually changes according to the amount and bioavailability of the Ca load, whether from food or a salt. When we compared the urinary excretion of Ca after the 1000 mg load from milk or the salt, there was no significant difference, which indicates that Ca was absorbed equally well from both forms of Ca. Kärkkäinen *et al*.^(^^14^^)^ reported increased urinary Ca excretion after a 400 mg dose of Ca as the salt compared with the same dose in milk. Green *et al*.^(^^11^^)^ reported no significant difference in urinary Ca excretion between milk fortified with milk Ca or calcium carbonate, and Martini & Wood^(^^26^^)^ reported no significant effect of Ca source on urinary Ca excretion using a similar load of Ca in orange juice, milk or as a salt in water. The above data therefore suggest that urinary Ca excretion is a function of Ca load and not sensitive to whether the Ca is provided as a salt or in a food. The kind of salt, however, may affect absorption and excretion with the lactate gluconate salt of Ca being more soluble, more absorbable and resulting in higher excretion compared with calcium carbonate or Ca from fortified milk^(^^2^^,^^14^^)^.

Ca supplements are widely used for the prevention or treatment of osteoporosis in postmenopausal women. However, some concern has been raised about the safety of using Ca supplements, as trials in healthy older women and in patients with renal impairment suggest that Ca supplementation may increase the risk of myocardial infarction, stroke and overall CVD^(^^13^^,^^30^^–^^32^^)^. Although this association remains controversial and the analyses have been strongly criticised, it has been hypothesised that a potential mechanism may involve the significant rise in serum ionised Ca (to the upper part – or over the normal range) that has been shown after a 1 g dose of Ca by Karp *et al*.^(^^2^^)^ and others^(^^13^^,^^22^^,^^30^^)^. The possibility exists that the higher circulating ionised Ca concentrations may affect vascular calcification, the function of the vascular cells and blood coagulation^(^^30^^–^^32^^)^.

The response in serum and ionised Ca to a Ca load over time has been shown to be different between Ca consumed in milk and that consumed as a Ca salt. The change in serum ionised Ca after a dose of a Ca salt was shown to be markedly higher (0·09 *v.* 0·075 mmol/l) and faster (peak at 2 h *v.* at 3·5–4 h) than the response to a similar dose of Ca in milk. In the present study we therefore have confirmed that the response in ionised Ca to a 1 g dose of Ca salt and 1 g Ca in milk is different.

In conclusion, the present data support the notion that the postprandial response to Ca from foods is different from that from a Ca salt, and that fortified milk can deliver relatively large doses of Ca without perturbing Ca homeostasis. This trial was done in young Asian women and the response to a load of Ca in this population may differ from that of older or postmenopausal women in other populations. The results should therefore be extrapolated with caution.
